# Is Metastatic Staging Needed for All Patients with Synchronous Bilateral Breast Cancers?

**DOI:** 10.3390/cancers16010017

**Published:** 2023-12-19

**Authors:** Geok Hoon Lim, Jing Xue Hoo, You Chan Shin, Rachel Zhi Ting Choo, Fuh Yong Wong, John Carson Allen

**Affiliations:** 1 Breast Department, KK Women’s and Children’s Hospital, Singapore 229899, Singapore; 2 Duke-NUS Medical School, Singapore 169857, Singapore; 3 Division of Radiation Oncology, National Cancer Centre, Singapore 168583, Singapore

**Keywords:** breast cancer, bilateral cancers, metastatic staging, systemic metastasis, synchronous cancers

## Abstract

**Simple Summary:**

Synchronous bilateral breast cancers are uncommon. While metastatic staging guidelines in patients with unilateral cancer are established, the indication for metastatic staging in patients with bilateral breast cancers remains unclear. This study aimed to retrospectively determine if all synchronous bilateral breast cancer patients need metastatic staging at diagnosis. This is the first such reported study, to the best of our knowledge. In our study, negative nodal status was predictive for negative metastatic staging results at diagnosis in patients with synchronous bilateral breast cancers. Nodal status alone, however, may not detect all cases with systemic metastasis. Hence, symptoms of systemic metastasis and metastatic nodal status could be used to determine the subgroup of synchronous bilateral invasive cancer patients who require metastatic staging. This finding could be validated in larger studies.

**Abstract:**

Background: Patients with bilateral breast cancers are uncommon and are associated with a poorer prognosis. While metastatic staging guidelines in patients with unilateral cancer were established, the indication of metastatic staging in patients with bilateral breast cancers is unclear. We aimed to determine which patients with synchronous bilateral breast cancers require metastatic staging at diagnosis. This is the first such reported study, to the best of our knowledge. Methods: A retrospective review of newly diagnosed synchronous bilateral invasive breast cancer patients at our institution was performed. We excluded patients with malignant phyllodes or no metastatic staging. Patients’ demographics and pathological and staging results were analysed to determine the group of bilateral breast cancer patients who required metastatic staging. Results: A total of 92 patients with synchronous bilateral invasive cancers were included. The mean age was 58 years old, and 64.1% had bilateral invasive ductal carcinoma. 23.9% had systemic metastasis. Nodal status was statistically significant for systemic metastasis on staging (*p* = 0.0081), with only three patients (3.3%) having negative nodal status and positive metastatic staging. These three patients, however, showed symptoms of distant metastasis. 92.3% of patients with negative nodes also had negative metastatic staging. Using negative nodal status as a guide avoided metastatic staging in 40.4% of all patients. Conclusions: Negative nodal status was the most predictive factor for no systemic metastasis on staging in patients with synchronous bilateral invasive breast cancers. Hence, metastatic staging could be reserved for patients with symptoms of systemic metastasis and/or metastatic nodes. This finding could be validated in larger studies.

## 1. Introduction

Patients with bilateral breast cancers are uncommon, with a reported incidence of 1.4–11.8% [1]. They can be categorised as either synchronous or metachronous bilateral cancer, depending on the time interval between the diagnosis of the primary and contralateral cancer. Synchronous bilateral breast cancer has been defined as a cancer exhibiting a time interval of 1 month [2] to 1 year [3] between the primary and contralateral cancer, depending on the definition used in various studies. Synchronous bilateral breast cancer also had a lower reported incidence rate of 1.6 per 10^5^ person-years at risk, while metachronous bilateral breast cancer (MBBC) had an incidence rate of 440 per 10^5^ person-years at risk [4].

In addition, synchronous bilateral breast cancer was also associated with a poorer prognosis [3]. While metastatic staging is reserved in unilateral breast cancers for patients with advanced breast cancer and/or symptoms suggestive of systemic metastasis [5], the role of metastatic staging in patients with synchronous bilateral breast cancer is unclear.

We aimed to determine which subgroup of patients with synchronous bilateral invasive breast cancer requires metastatic staging at diagnosis. To the best of our knowledge, this is the first such reported study.

## 2. Materials and Methods

Synchronous bilateral invasive breast cancer patients who were newly diagnosed at KK Women’s and Children’s Hospital from 1 September 2005 to 30 June 2022 were included in this retrospective study. Patients were considered to have synchronous bilateral breast cancer at contralateral cancer diagnosis when the contralateral cancer was diagnosed ≤1 month following the diagnosis of the primary cancer. We excluded patients with malignant phyllodes, bilateral pure ductal carcinoma in situ (DCIS), patients with ipsilateral invasive cancer and contralateral pure DCIS, and patients with no metastatic staging.

At our institution, metastatic staging, consisting of Computed Tomography (CT) and a bone scan, was performed routinely on breast cancer patients with advanced disease and typically on patients with nodal involvement. For patients who had contraindications to a CT scan, a chest X-ray and hepatobiliary ultrasound were performed instead. In some cases, metastatic staging may also be performed in patients with early breast cancer, based on the treating physician’s discretion.

In patients with indeterminate findings on the initial metastatic staging, further investigations or follow-up was conducted to characterise these findings. In this study, for indeterminate findings relying on follow-up for further characterisation, lesions were considered benign if there was no progression or recurrence after 2 years of follow-up. As a result, patients with indeterminate lesions on initial metastatic staging and a follow-up period shorter than 2 years were excluded from this study. Similarly, in patients with incomplete metastatic staging at initial diagnosis, a follow-up period of 2 years was used to determine metastatic staging status.

Demographics and histological characteristics of bilateral cancers and metastatic staging outcomes were collected from a prospectively maintained database. Pathological characteristics were based on surgical histology. A patient was defined as node positive if there was histological confirmation. In stage IV patients with no surgery or in patients with neoadjuvant chemotherapy, characteristics such as grade, estrogen receptor (ER) and progesterone receptor (PR) etc., were obtained from the biopsy. In these patients, no tumour size was recorded since stage IV patients usually do not undergo surgery, while in patients with neoadjuvant chemotherapy, the tumour size was not well reflected on surgical histology. These characteristics were then analysed between patients with systemic metastasis versus those without, on metastatic staging, in order to determine the risk factors in synchronous bilateral breast cancer patients who required metastatic staging.

### Statistical Analysis

Fisher’s exact test was employed to compare the categorical variable frequency outcomes between synchronous bilateral breast cancer patients with systemic metastasis versus those without on metastatic staging. For comparisons among patients, the worst pathological feature for each patient among the bilateral cancers, such as the higher grade or larger tumour size, was selected. For patients with unknown grade status on either side, the classification was recorded as unknown unless there was a known grade III tumour on either side. Then, it would be categorised as grade III despite the unknown grade on the other side. The patients were categorised as node-positive when either cancer showed nodal positivity. For hormonal and human epidermal growth factor receptor 2 (HER2) status, the patients were grouped into positive/positive, positive/negative and negative/negative based on the receptor status for the dominant and contralateral cancer. If the receptor status on either side was unknown, these patients were classified as unknown and excluded from analysis. *p* < 0.05 was defined as statistically significant. SAS statistical software (v9.4) was used for the analysis.

This study was approved by the SingHealth Centralised Institutional Review Board (CIRB Ref: 2019/2419), and patients’ informed consent was waived.

## 3. Results

A total of 3835 invasive breast cancer patients were diagnosed during this study period. Of these patients, there were 156 histologically confirmed synchronous bilateral breast cancers. Of these patients, 1 patient had synchronous bilateral DCIS, 61 patients had ipsilateral invasive cancer and contralateral DCIS, and another had ipsilateral malignant phyllodes and contralateral invasive breast cancer. Hence, 93 (2.4%) patients had synchronous bilateral invasive cancers. However, 1 patient did not have metastatic staging and was excluded, leaving 92 patients for analysis.

The mean age of the included cohort was 58 years (range: 30–86), and 23.9% of patients had systemic metastasis at staging. The predominant histology was invasive ductal carcinoma, with 64.1% having invasive ductal carcinoma bilaterally. A total of 5.4% of the patients in the cohort had bilateral invasive lobular cancer. For the dominant cancer, 15 patients had other histology besides invasive ductal carcinoma or invasive lobular cancer. These histologies included six mucinous cancers, three metaplastic cancers, three papillary cancers, one invasive micropapillary/ductal cancer, one tubular cribiform carcinoma and one invasive apocrine carcinoma. For the contralateral cancer, the other histologies included seven mucinous cancers, two invasive ductal/tubular cancers, one mixed invasive ductal and lobular carcinoma and one invasive micropapillary/ductal cancer. 

The majority of patients had hormone receptor status positivity and HER2 receptor status negativity on both sides (Table 1). Mean tumour size for dominant and contralateral cancer was 37.2 mm (range: 12–157) and 15.5 mm (range: 1–100), respectively.

Overall, 50 (54.3%) patients had histologically proven nodal involvement (Table 2). Of these, 22 (23.9%) had nodal positivity for both breast cancers. Three patients were classified as having unknown nodal status, as there was no histological confirmation of the lymph node status although there were radiologically abnormal lymph nodes. In view of their stage IV status, these patients did not undergo nodal biopsy.

On statistical analysis, nodal status was statistically significant as a predictor for systemic metastasis on staging (*p* = 0.0081) (Table 2). A total of 39 patients had negative nodal status. Of these 39 patients, 36/39 (92.3%) also had negative staging results. As a result, negative nodal status was predictive of negative metastatic staging outcome and could be used as a guide to exclude patients from unnecessary metastatic staging. Using negative nodal status as a criterion for not performing metastatic staging could avoid the use of metastatic staging in 36/89 (40.4%) of patients in our study.

On the other hand, three patients (3.3%) had negative nodal status and positive metastatic staging results. These three patients all had symptoms of distant metastasis. Hence, symptoms of systemic metastasis could also be used with nodal status to determine the need for metastatic staging.

## 4. Discussion

In our cohort, 2.4% of patients had synchronous bilateral invasive cancers. Of the eligible patients, 23.9% had systemic metastasis at staging. Negative nodal status was statistically significant for no systemic metastasis at staging. In patients with distant metastasis on staging but no nodal involvement, all displayed symptoms of distant metastasis. As a result, metastatic nodal status and symptoms of metastatic disease could be used as a guide for the selection of synchronous bilateral invasive breast cancer patients for metastatic staging. This is the first such study, to the best of our knowledge.

Synchronous bilateral breast cancer is an uncommon entity, and our prevalence rate of synchronous bilateral breast cancer was similar to that reported in the literature [1,6]. Our patients’ histological features were also consistent with the literature, with invasive ductal carcinoma and ER-positive/HER2-negative molecular subtype being the most common histology [7,8] in synchronous bilateral breast cancers. While invasive lobular carcinoma was associated with multicentric and bilateral breast cancers [9], only 5.4% of the patients in our cohort had bilateral invasive lobular carcinoma. For invasive lobular carcinoma, there were conflicting reports on its clinical outcomes. While some reported no worse clinical outcomes than that of invasive ductal carcinoma [10], there were other contradictory reports that suggested a higher risk of distant metastasis after long-term follow-up [11]. As for the other histologies, invasive micropapillary cancer and metaplastic cancer are rare subtypes [12,13] that have been associated with worse pathological features, though they have not been specifically associated with bilateral breast cancer. Invasive micropapillary cancer more often exists in a mixed form, as witnessed in our study. It tends to be associated with larger tumour size, higher grade and a higher risk of nodal metastasis [12]. Despite this, its incidence of systemic metastasis was reported to be low at 3.6% in a large cohort of patients with invasive micropapillary cancer [14]. Metaplastic cancer, on the other hand, is a heterogenous group of cancers that were also associated with unfavourable pathological features. Unlike invasive micropapillary cancers that are more commonly associated with hormonal receptor and HER2 positivity, metaplastic cancers tend to be associated with triple-negative phenotypes, with the majority having an inferior outcome [15]. In our study, however, the histological subtype was not one of the statistically significant factors for predicting systemic metastasis at staging.

Synchronous bilateral breast cancer has been associated with a poorer prognosis [3,6,16,17] with a higher propensity for distant metastasis compared to unilateral cancers [18,19]. As a result, it remains unclear whether metastatic staging should be advocated for all patients with synchronous bilateral breast cancer or reserved for a subgroup of patients with synchronous bilateral breast cancer.

For unilateral breast cancers, current international guidelines (American Society of Clinical Oncology (ASCO), National Comprehensive Cancer Network (NCCN), European Society for Medical Oncology (ESMO), etc.) do not routinely recommend metastatic staging in asymptomatic early-stage breast cancer patients unless there are indications such as symptoms or signs of metastasis [20,21,22]. This is because the yield of detecting systemic metastasis in this group of patients is very low [21,22]. It was reported that the possibility of detection of systemic metastasis in asymptomatic stage I and II patients was 0% to 5.1% and 0–5.5%, respectively [20]. This low yield was also demonstrated in asymptomatic stage II breast cancer patients regardless of their molecular subtypes [23].

In addition, metastatic staging can also result in false positive findings, which could lead to further investigations. Patients with metastatic staging could have indeterminate findings in 7.1–12.1% of patients [24,25]. This warranted further investigations and was subsequently proven to be non-metastatic in 94.6–96.2% of cases. Unnecessary metastatic staging could also lead to increased patient anxiety and financial burden [26]. As a result, metastatic staging should only be performed when indicated.

While there are established guidelines for the role of metastatic staging in patients with unilateral breast cancer, the indication for metastatic staging in patients with synchronous bilateral breast cancer is unclear. Our study showed that nodal involvement was statistically associated with the risk of distant metastasis on staging scans. This was consistent with the literature, which showed that locoregional lymph node involvement was often predictive of potential distant metastasis in patients with breast cancer [27,28]. Nodal involvement is a known poor prognostic factor for breast cancer [29] and is associated with an increased risk for distant metastasis [30]. Our findings were also supported by Gerber et al. [24], who reported a higher frequency of detection of systemic metastasis at staging with a higher nodal burden. This is also concordant with the staging guidelines for unilateral cancers, which advocated the use of staging scans for advanced breast cancers. As a result, nodal status could be used to guide the use of metastatic staging in patients with synchronous bilateral breast cancer.

While nodal status was predictive of systemic metastasis, patients with negative nodal status could still have systemic metastasis [31]. Conversely, not all patients with positive nodal status would have systemic metastasis; hence, the relationship between nodal status and systemic metastasis is not unequivocal [32]. It was reported that about one-third of breast cancer patients with a negative nodal status could still develop systemic metastasis, while one-third of breast cancer patients with a positive nodal status do not develop systemic metastasis at all [32]. Despite this, nodal status remained the most predictive factor for systemic metastasis at staging in our study. Though it may not detect all cases with systemic metastasis, it could guide the clinicians on the appropriate use of metastatic staging. This was especially useful when there was a negative nodal status since using this predictive factor avoided the use of metastatic staging in 40.4% of patients in our study.

In our study, though three patients had no nodal metastasis, they had systemic metastasis at staging. In these cases, they all had symptoms suggestive of systemic metastasis. As a result, symptoms of systemic metastasis could also be used as another predictive factor, in addition to nodal status, to identify the group of patients who will benefit from metastatic staging.

In our study, patients with pure bilateral or ipsilateral DCIS were excluded because DCIS is a preinvasive disease [33], and the risk of systemic metastasis was reported to be very low at about 0.5% [34]. Instead of DCIS resulting in systemic metastasis, this risk of systemic metastasis could be attributed to an occult invasive component that was not recognized at surgical histology [35] or to a subsequent invasive recurrence.

This is the first reported such study, to the best of our knowledge. The strengths of this study included comparable histological parameters for synchronous bilateral breast cancers, as reported in the literature. Metastatic staging results were available for the majority of our patients. Most patients underwent nodal biopsy for histological confirmation if there was evidence of abnormal lymph nodes on imaging.

Limitations of this study included its retrospective nature, and there may be selection bias of patients who underwent metastatic staging. It had a small sample size, which was not surprising in view of the low prevalence of synchronous bilateral breast cancers. Our findings could be validated in larger prospective studies in the future.

## 5. Conclusions

Nodal status was the most predictive factor for systemic metastasis in patients with synchronous bilateral invasive breast cancers, with negative nodal status likely to result in a negative metastatic staging outcome. Despite nodal status being the most predictive factor, it could not detect all patients with systemic metastasis on staging. As a result, symptoms of systemic metastasis could be used, in conjunction with nodal status, to guide the clinicians on the use of metastatic staging for this group of patients with a known poorer prognosis. This finding could be validated in larger prospective studies in the future. 

## Figures and Tables

**Table 1:** Characteristics of patients with synchronous bilateral invasive breast cancers.

*n* = 92	Dominant *n* = 92 (%)	Contralateral *n* = 92 (%)
Age at diagnosis/years		
<50	26 (28.3)	
≥50	66 (71.7)	
Histological features		
IDC	68 (73.9)	73 (79.3)
ILC	9 (9.8)	7 (7.6)
Others	15 (16.3)	11 (12.0)
Unknown	0 (0)	1 (1.1)
Grade		
I	15 (16.3)	25 (27.2)
II	41 (44.6)	40 (43.5)
III	29 (31.5)	16 (17.4)
unknown	7 (7.6)	11 (12.0)
Tumour size/mm		
≤20	11 (12.0)	44 (47.8)
>20–50	36 (39.1)	9 (9.8)
>50	8 (8.7)	1 (1.1)
unknown	37 (40.2)	38 (41.3)
ER		
positive	75 (81.5)	82 (89.1)
negative	17 (18.5)	9 (9.8)
unknown	0 (0)	1 (1.1)
PR		
positive	67 (72.8)	75 (81.5)
negative	25 (27.2)	16(17.4)
unknown	0 (0)	1 (1.1)
Her2		
positive	20 (21.7)	10 (10.9)
negative	72 (78.3)	79 (85.9)
unknown	0 (0)	3 (3.3)
Nodal status		
positive	50 (54.3)	22 (23.9)
negative	39 (42.4)	67 (72.8)
unknown	3 (3.3)	3 (3.3)

**Table 2:** Comparison of metastatic staging results in patients with synchronous bilateral invasive cancers.

*n* = 92	Patients with Positive Metastatic Workup *n* = 22 (%)	Patients with Negative Metastatic Workup *n* = 70 (%)	*p* Value
Age at diagnosis/years			0.4166
<50	8 (36.4)	18 (25.7)	
≥50	14 (63.6)	52 (74.3)	
Histological features			0.1768
IDC/IDC	15 (68.2)	44 (63.8)	
ILC/ILC	3 (13.6)	2 (2.9)	
IDC/others	3 (13.6)	19 (27.5)	
Others	1 (4.5)	4 (5.8)	
Unknown	0	1	
Grade *			1.0000
I	1 (5.6)	6 (9.2)	
II	9 (50.0)	33 (50.8)	
III	8 (44.4)	26 (40.0)	
unknown	4	5	
Tumour size/mm **			1.0000
≤20	0 (0.0)	11 (20.4)	
>20–50	1 (100.0)	35 (64.8)	
>50	0 (0.0)	8 (14.8)	
unknown	21	16	
ER			0.3294
Positive/positive	15 (68.2)	56 (81.2)	
Negative/positive	5 (22.7)	10 (14.5)	
Negative/negative	2 (9.1)	3 (4.3)	
unknown	0	1	
PR			0.0595
Positive/positive	12 (54.5)	49 (71.0)	
Negative/positive	4 (18.2)	15 (21.7)	
Negative/negative	6 (27.3)	5 (7.2)	
Unknown	0	1	
Her2			0.2660
Positive/positive	3 (14.3)	3 (4.4)	
Negative/positive	4 (19.0)	12 (17.7)	
Negative/negative	14 (66.7)	53 (77.9)	
Unknown	1	2	
Nodal status #			0.0081
positive	16 (84.2)	34 (48.6)	
negative	3 (15.8)	36 (51.4)	
Unknown	3	0	

* Higher grade recorded; ** larger size recorded; # recorded as positive if lymph node was positive on either side.

## Data Availability

The data are available from the corresponding author upon request.
